# Promoting Service Leadership Qualities and Well-Being among University Students through an Online Course during COVID-19 Pandemic

**DOI:** 10.3390/ijerph18158162

**Published:** 2021-08-02

**Authors:** Xiaoqin Zhu, Daniel T. L. Shek, Cathy H. M. Chan

**Affiliations:** Department of Applied Social Sciences, The Hong Kong Polytechnic University, Hong Kong, China; xiaoqin.zhu@polyu.edu.hk (X.Z.); cathy.hm.chan@polyu.edu.hk (C.H.M.C.)

**Keywords:** online learning mode, blended learning, service leadership, course evaluation, COVID-19

## Abstract

The COVID-19 pandemic is a great challenge to leadership education in universities. Although previous findings provide support for the effectiveness of online learning, the impact of online leadership course on students’ learning outcomes and well-being has not been well documented. Using objective outcome and subjective outcome evaluation strategies, the present study examined students’ perceived qualities and effectiveness of an online credit-bearing service leadership course adopting asynchronous mode (primarily online learning) and synchronous mode under COVID-19. Regardless of teaching modes, the subject yielded positive impacts. Specifically, pretest-posttest (N = 228) showed that there were positive changes in students’ service leadership qualities, life satisfaction and psychological well-being. For students’ perception of the course (N = 219), results indicated that most students were positive in their learning experience and satisfied with course design, lecturer quality and the benefits of the course to their development. Students’ changes and subjective perceptions were positively correlated, but with a low effect size. The findings reflected that online service leadership course adopting asynchronous or synchronous mode was effective, and students were positive about their learning experience.

## 1. Introduction

Since the first appearance of the novel coronavirus disease (COVID-19), over 156 million students from all age groups have been affected due to the closing down of educational institutions [1]. Governments all over the world have imposed lockdown and social distancing measures to avoid face-to-face interactions to curb the spread of the disease. The closure of educational institutions greatly impacts education practices and the most obvious change is turning face-to-face lecturing in physical classrooms into online learning [2]. Past studies showed mixed results on the benefits and challenges of online learning [3,4]. The learning environment under COVID-19 is much more complex than before because it is an overnight change of physical classrooms into e-classrooms in a massive manner, and online learning is the only available option under strict restrictions on social distancing. Without alternative modes of instruction, reassuring students and satisfying their learning needs at different levels and stages in a digital environment is a challenge to education institutions [5]. Students are suffering from fatigue caused by both, the seemingly endless disease with the prevention measures and the rapid changing living and learning circumstances, and their learning and mental health status have become a matter of great concern [6,7].

The current study reports the effectiveness and students’ perception of online “Service Leadership” courses in a university in Hong Kong under COVID-19 using different evaluation methods. Although past studies showed that classroom teaching of service leadership has positive impacts on students’ leadership quality development and well-being [8], few related studies have been conducted under COVID-19 pandemic. Given that the face-to-face “Service Leadership” course has intensive interactions to facilitate students’ deep learning, the online environment poses challenges to the delivery of the course, and course effectiveness becomes a field for exploration. Moreover, students’ perceptions of the course should be taken into account in course evaluation since their feedback provides valuable information for course refinement. The findings of this study would provide insights into the development of online leadership programs in higher education.

### 1.1. Service Leadership Education and Its Effectiveness

The contribution by service industries to the world gross domestic product (GDP) kept increasing over the past two decades [9], and it serves as a major driving force for economic development. Conceptually, services refer to multiple economic activities that involve “the provision of human value added in the form of labor, advice, managerial skill, entertainment, training, intermediation and the like” ([10], p. 7). Service economy is characterized by complex human interactions in service provisions and shared decision-making, as well as empowerment in more decentralized organizational structures [11,12]. The conventional concepts and the nature of leadership have changed along with the economic shift. Traditional leadership models emphasize efficient manipulation of employees and strong competent leaders. In a service economy, leadership is considered a relational and ethical process [13] and a balance between alignment and empowerment [14], where effective leaders are expected to show not only professional competence but also communication skills, ethical traits, service orientation, consideration, empathy and caring disposition [15].

The service leadership model (SLM) is a good example of models that address the needs of the service economy. According to the model [16], service leadership is about “satisfying needs by consistently providing quality personal service to everyone one comes into contact with, including one’s self, others, groups, communities, systems, and environments” (p. 217). This model upholds the belief that “everyone is (and can be) a leader” regardless of their position and encourages continuous commitment to make self-improvement [16], and highlights the importance of the three Cs of leadership qualities: competence, character and care. Different from other propositions that regard leadership as an instrument of profits (e.g., top-down leadership) or neglect the personal needs of the leaders (e.g., servant leadership), the SLM sees ethical satisfaction of one’s own needs as a prerequisite of continuous self-development and sustainable leadership [16,17].

In response to the shift in the economic structure, there is a need for service leadership education that nurtures leadership qualities within university students [18,19]. Since the SLM represents a positive response to the shift in the global economy and it emphasizes the development of positive values and traits, it has been promoted in guiding service leadership education in Hong Kong [15]. Apart from providing students with an understanding of the related concepts and skills in leadership and cultivating service-oriented values and beliefs [20], service leadership courses also facilitate the development of service leadership attributes, such as intrapersonal and interpersonal competencies, continuous self-improvement and self-reflection, morality and care [15,16]. 

Indeed, service leadership education targets the holistic development of students. When students have new experiences and experience an improvement in leadership qualities, such as resilience, self-understanding, emotional competence, they may then have a sense of growth in both cognitive and affective domains, which may lead to an increase in positive functioning (psychological well-being) and positive feelings (subjective well-being) [17]. For example, Zhu and Shek [8] investigated the effectiveness of the credit-bearing “Service Leadership” course offered to university students and the results showed an improvement in both leadership and well-being development among the students. Other studies yielded similar results, suggesting that curriculum-based service leadership education is effective in promoting students’ service leadership development and well-being [17,21,22].

### 1.2. Learning under COVID-19

Due to the COVID-19 pandemic worldwide, traditional face-to-face lectures were suspended and moved online. Online learning can be defined as “learning experiences in synchronous or asynchronous environments using different devices with internet access. In these environments, students can be anywhere to learn and interact with instructors and other students” ([23], p. 302). Divergent results have been reported regarding the impacts of online learning. Benefits of learning online include flexible use of time and location, wide availability of content, interactive and self-direct and higher levels of engagement, satisfaction, as well as achievement [24,25], but the benefits depend on factors such as digital competence of the users, equality of resources, technology, assessment and workload [26].

The outbreak of COVID-19 with the closure of schools has led to the massive adoption of online learning. Institutes, teachers and students faced different kinds of challenges in online learning. Teachers had to adjust their pedagogical approach to tackle the new situations and maintain teaching quality. To stimulate students’ learning, interactive, student-centered and group-based lesson designs in the e-classroom were suggested [27]. Students had no option but to stay at home and learn online with their devices. Besides, face-to-face support from lecturers and peers was no longer available. Recent studies explored students’ learning during COVID-19 and showed that students faced various problems related to digital exclusion, such as limited internet access and an unfavorable learning environment at home [28]. In Hong Kong, a study on the online learning experience under COVID-19 found students faced a decrease in learning effectiveness, studying time and efficiency, and they perceived an increase in studying pressure [29]. However, in the context of service-learning, there is evidence showing that the online service-learning activities under COVID-19 promoted the positive development of both service providers and service recipients [30].

In addition to the COVID-19 pandemic, the impact of the civil unrest in 2019 may still undergo in Hong Kong. Studies have shown that Hong Kong people’s life quality and mental health have been adversely affected by the movement from different perspectives [31]. While the stress and anxiety brought by the social tension and uncertainty were not yet digested, the “hit” of the pandemic with the social isolation due to the disease further increased the feelings of anxiety, loneliness, depression and perhaps post-traumatic stress disorder. Obviously, the mix of sudden changes in learning and continuous tense social atmosphere has exerted additional impacts, all of which may affect their learning and lower their well-being.

### 1.3. Research Gaps

The benefits of traditional classroom service leadership education on leadership development and well-being among university students were examined and reported in previous studies [8,17]. For example, there are studies examining students’ perception toward classroom instructed service leadership education and found students were positive to subject content, teachers, and course benefits [22]. Yet, the effectiveness of online service leadership education and students’ subjective feelings towards online learning under the circumstance of the pandemic remain unknown [30]. When the instruction becomes more hybrid, it is important to understand course effectiveness and subjective learning experience from students’ perspective. 

In addition, previous studies have not investigated the relationship between leadership course effectiveness (i.e., students’ changes after taking the subject) and students’ perceptions of the subject (i.e., their satisfaction with the subject). The model of training evaluation assumes that evaluation at different levels is positively correlated with each other, such as learners’ satisfaction and their learning achievement [32]. This conjecture is supported by some empirical evidence in face-to-face learning environment [33]. However, there are a few studies on online learning yielded negative or no significant correlations between these two domains. For instance, in the meta-analysis, Ebner and Gegenfurtner [24] identified a negative association between satisfaction level and learning gain in three different teaching modes. Nevertheless, a positive linkage between satisfaction and online learning achievement was supported by the social interaction during the lesson, no matter among peers or between students and lecturers [34,35,36].

### 1.4. The Present Study

The aim of this study was to examine the effectiveness and students’ perception of an online credit-bearing “Service Leadership” course in one university under the COVID-19 pandemic in Hong Kong. The correlation between course effectiveness and students’ perception was also investigated. A single group pretest-posttest design was used to examine course effectiveness in terms of students’ changes in service leadership qualities and well-being attributes. Subjective outcome evaluation through a client satisfaction approach was used in measuring students’ perception towards the online courses. These two evaluation methods are frequently utilized in the evaluation of prevention or intervention programs in the social sciences fields [33,37].

To sum up, the following three research questions would be addressed:

Research Question 1: “Do students taking online ’Service Leadership’ course show significant improvements after completing the course?” Based on previous studies [8,17], we had the following hypotheses.

**Hypothesis** **1a** **(H1a):** *Students would have improvements in posttest in comparison to pretest scores in service leadership qualities*.

**Hypothesis** **1b** **(H1b):** *Students would have improvements in posttest in comparison to pretest scores in well-being*.

Research Question 2: “What are the students’ perceptions of the online ’Service Leadership” course?’

**Hypothesis** **2a** **(H2a):** *Student would have positive perceptions in subject content*.

**Hypothesis** **2b** **(H2b):** *Students would have positive perceptions in lecturer quality*.

**Hypothesis** **2c** **(H2c):** *Students would have positive perceptions in benefits of the online course*.

Research Question 3: “How are the objective learning outcomes associated with students’ subjective perceptions of the online course?” Although there are mixed findings in the literature [24,34,35,36], we found that objective outcomes were positively related to subjective outcomes [33]. Hence, we had the following hypothesis.

**Hypothesis** **3** **(H3):** *Change in the objective outcomes would be positively associated with students’ subjective perceptions of the course*.

## 2. Materials and Methods

### 2.1. Overview of the “Service Leadership” Subject during COVID-19 Pandemic

The service leadership subject included 13 lectures covering key concepts and beliefs of the service leadership model (SLM), including unique features of service economy, core beliefs such as “everyone can be a leader”, analysis of SLM in comparison to other leadership models and important qualities that determine effective leadership under service economy, including generic competencies (e.g., social skills and self-leadership), character (e.g., character strength and Confucian virtues) and care (e.g., active learning). In addition to the theoretical knowledge, the course also included rich real-life examples and activities in multiple formats (e.g., role play, debate, and reflection) to facilitate students’ deep learning and active application of the knowledge in their daily life and future career. 

The original service leadership subject adopted the pedagogy centering on students’ in-classroom learning, characterized by multiple interactive in-class activities (sharing, drawing, role-play and debate), self-reflection exercises, collaborative learning (e.g., group discussion, group sharing and group presentation) and critical thinking practice (e.g., critical evaluation of leadership qualities from multiple perspectives). It is expected that by “experiencing, reflecting, thinking and acting”, students can be more engaged and perform deep learning [38,39].

In Semester 2 and Semester 3 of 2019–2020, in order to maintain social distance under the COVID-19 pandemic, all classroom teaching was changed to online teaching. Regarding the “Service Leadership” course, two modes of online teaching were adopted. The first mode was a combination of ten asynchronous e-learning lectures and three synchronous online lectures (asynchronous mode hereafter). Each asynchronous e-learning lecture included several online lecturing videos covering course content under the same topic. The length of the videos ranged between three to eighteen minutes with an average duration of seven minutes. In addition to taped lectures, reflective activities, online group discussion, online discussion board and online chat sessions were also incorporated as online activities to enhance interactions among students. Students were required to finish each e-learning lecture within the respective week. This arrangement let students have a flexible study schedule and they could re-visit learning materials anytime they wanted to. The three synchronous online lectures were the first, sixth and thirteenth lectures, which were intended to give students introduction, feedback to online activities, opportunities for real-time interactions and periodical wrap-up. 

The second mode was that all the 13 lectures were delivered by lecturers through synchronous online teaching and in-class activities were adapted to online version (synchronous mode hereafter). We used a real-time conferencing tool entitled “Blackboard Collaborate Ultra” to conduct synchronous online lectures where students attended the lectures remotely at the scheduled class time and participated in interactive activities online. In these lectures, in addition to synchronous lecturing, students and teachers could have real-time interaction in multiple ways, including both individual and group activities. For individual activities, students could raise questions or respond to teachers’ questions by sharing audio or typing in the chat box. In addition, teachers could use a whiteboard to let students type or draw their opinions simultaneously. Teachers could also use polling function or external tools such as Mentimeter to do real-time voting. For group activities, students were randomly assigned to different groups at the first lecture (around 8–10 students per group) and each group would complete group discussion activities in their respective “Group Collaborate Ultra” and some groups were invited to share in the main classroom after group discussion. 

While the teaching approaches, especially how students and teachers interacted, were different between the asynchronous and the synchronous modes, the course materials and assessment requirements, as well as lecturers, were the same for the two delivery modes.

### 2.2. Participants and Procedures

In Semester 2 of the 2019–2020 year, there was one class of service leadership through the asynchronous mode in thirteen weeks. In Semester 3 (i.e., the summer course), the subject was offered intensively in seven weeks—two classes using the two modes, respectively. Participants in the present study were students in these three classes. They were invited to complete the pretest questionnaire within one week before the first lecture and complete the same online questionnaire as the posttest within one week upon completing the last lecture. Students also completed an online subjective outcome evaluation (SOE) form at the end of the last lecture to express their perceptions of the course they had completed. An online information sheet was provided to students to explain principles of confidentiality, voluntary participation, no release of individual identity information and the usage of data only for educational and research purpose. Students indicated their consent in an online form before responding to the questionnaires. Ethical approval was gained from the “Human Subjects Ethics Sub-committee” (HSESC) at the authors’ affiliated university.

A total of 75, 79 and 74 students were matched in the pretest-posttest evaluation in the three classes, respectively (see Table 1). Among these participants (total N = 228), 101 (44.30%) were males and 127 (55.70%) were females, and the mean age was 19.97 years with a standard deviation of 1.92. Completed SOE form were collected from 54, 79 and 86 (total N = 219) students in different courses, respectively (see Table 1). Additionally, 207 students completed both the pretest-posttest questionnaires and the SOE form. 

### 2.3. Measures

The pretest and posttest questionnaires included measures of (1) service leadership qualities indexed by “knowledge”, “attitude” and “behavior”, (2) subjective well-being indexed by “life satisfaction” and (3) psychological well-being indexed by “positive youth development (PYD) attributes”. The present study also used the SOE form. All the measures are outlined in the below sections with details.

Knowledge about service leadership was assessed by 40 multiple-choice questions covered in the “Service Leadership Knowledge Scale” (SLK). These questions were related to different service leadership concepts covered in the lectures, such as the social and economic background of the service economy, key determinants of effective service leadership and beliefs about self-leadership [40]. One or zero points were given to each correct or incorrect answer, respectively. Thus, the theoretical range of SLK total score is 0 to 40. This scale has undergone a systematic validation procedure [40] and showed good reliability [8]. In this study, the internal consistency of the SLK was also good (see Table 2).

A 23-item short version of the “Service Leadership Attitude Scale” (23-SLA) was used in the present study to measure participants’ attitude regarding service leadership. The 23-SLA was derived from the 46-item full version, which has also been validated through a series of studies including content validation and comprehensive factorial validation [41]. The full scale possessed good validity and reliability and included eight subscales pertinent to different aspects of attitude, such as “people orientation”, “being ethical role model”, “frequent self-reflection” and so forth [41]. The short version comprised half of the items that showed highest factor loadings. Participants gave their responses using a 6-point scale (“1” = “strongly disagree”, “6” = “strongly agree”) on each item (e.g., “everyone has the potential to be a leader”, “a good leader listens to his/her subordinates’ views”, and “a leader should closely examine his/her own thoughts and behavior”). As shown in Table 2, the 23-SLA had good internal consistency across all assessment occasions in the present study.

The short version of the “Service Leadership Behavior Scale” (19-SLB), which included half of the items in the 38-item full version (i.e., 38-SLB), was employed to measure leadership behavior. The 38-SLB, which consisted of six subscales (e.g., “resilience”, “self-improvement and self-reflection”, “problem-solving”, etc.), showed adequate validity and reliability [42,43]. Similar to the attitude scale, the short version of the behavior scale was formed by selecting items with highest factor loadings. Sample items included “I often try my best to help other people to overcome difficulties”, “I learn through reflecting on my experiences” and “I am able to think independently”. Participants rated each item from “1” (“strongly disagree”) to “6” (“strongly agree”). The 19-SLB showed good reliability in previous research [8] and the present study (see Table 2).

The Chinese version of the “Satisfaction with Life Scale” (C-SWLS) was adopted to measure participants’ life satisfaction, which indicated subjective well-being in the present study. The C-SWLS assessed respondents’ cognitive evaluation on their life quality through five items (e.g., “In most ways, my life is close to my ideal.” and “The conditions of my life are excellent.”) from “1” (“strongly disagree”) to “6” (“strongly agree”). With good reliability, this scale has been widely employed among Chinese people [37,44]. In our study, the scale’s Cronbach’s α estimates were higher than 0.85 in all cases (see Table 2).

Similar to previous evaluation research [8,17], a 31-item short version of the “Chinese Positive Youth Development Scale” (CPYDS), which included 10 out of 15 original subscales, was used to assess participants’ PYD attributes as a measure of psychological well-being. The ten PYD attributes can be grouped into three clusters, including (1) “Cognitive-behavioral competence” measured by “cognitive competence”, “behavioral competence” and “self-determination”; (2) “Positive identity” measured by “clear and positive identity” and “belief in future”; and (3) general PYD qualities assessed by “social competence”, “emotional competence”, “moral competence”, “spirituality” and “resilience”. Participants rated all items on a 6-point scale (“1” = “strongly disagree”, “6” = “strongly agree”). We calculated average scores for the three clusters as well as the total average score across all items. Cronbach’s α values of all subscales were greater than 0.80 in this study (see Table 2). 

The 38-item SOE form used in the present study measured students’ perceptions of the “Service Leadership” courses using the online teaching mode. Specifically, three subscales assessed three aspects of the subject, respectively, including (1) “course content” (10 items, e.g., “the content design of the curriculum is very good”), (2) “lecturer performance” (10 items, e.g., “the lecturers showed good professional attitudes”) and (3) “course benefits” (18 items, e.g., “it has strengthened my self-confidence”). A 5-point scale was used (“1” = “strongly disagree”, “5” = “strongly agree”). Cronbach’s α values of the subscales varied between 0.90 and 0.97 in the present study. 

### 2.4. Data Analysis 

Data analysis involved three steps using SPSS 26.0 (IBM Corp., Somers, NY, USA). First, students’ pretest and posttest scores on PYD attributes, life satisfaction, and service leadership qualities were compared through “repeated-measures general linear model” (GLM hereafter). In GLM, while test scores were dependent variables, testing time (i.e., pretest vs. posttest) was the independent variable. For PYD attributes and service leadership qualities which included multiple indicators, the Bonferroni procedure was employed in detecting the potential omnibus time effect. If the multivariate effect was statistically significant, we further performed univariate analyses to compare test scores in each measure of PYD attributes and leadership qualities. We first tested whether the online course mode (asynchronous vs. synchronous) and course time (Semester 2 with a duration of 13 weeks vs. Semester 3 with a duration of 7 weeks) had any main effects or interactions with testing time on the dependent variables. If there were no significant main or interaction effects of course mode and course time, data were analyzed in terms of both separate samples collected in each course and whole sample combining all data, with time (pretest versus posttest) as the main factor. 

Second, numbers and percentages of positive responses in each item of the SOE form were checked through descriptive statistics. Participants’ response profiles were also examined based on each separate course and the whole sample. Besides, a multivariate GLM analysis was conducted to investigate whether there were any differences in students’ subjective evaluation for the three courses. 

Finally, correlational analyses were conducted based on the whole matched sample (N = 207) to check whether student posttest scores and changes in well-being and leadership qualities after completing the course were significantly correlated with their subjective perceptions on different aspects of the course. 

## 3. Results

### 3.1. Students’ Changes 

Results showed that online course mode (asynchronous vs. synchronous) and the course time (Semester 2 vs. Semester 3) did not have any significant main effects (F ranged between 0.003 and 2.29, ps > 0.05) or interactions with other factors (F ranged between 0.12 and 2.09, ps > 0.05) on students’ changes. 

As shown in Table 2, for all the three courses, significant omnibus time effects were observed for PYD attributes (F ranged between 4.72 and 7.16, ps < 0.001, η^2^_p_ ranged between 0.17 and 0.24), life satisfaction (F ranged between 10.14 and 18.88, ps < 0.01, η^2^_p_ ranged between 0.12 and 0.21) and service leadership qualities (F ranged between 5.78 and 6.94, ps < 0.01, η^2^_p_ ranged between 0.20 and 0.22). 

Results of follow-up univariate analyses are also depicted in Table 2. Findings revealed that except general PYD qualities, positive changes were found in other PYD measures (F ranged between 4.35 and 21.69, ps < 0.05, η^2^_p_ ranged between 0.06 and 0.20) after students took the asynchronous online course in the 2nd Semester. For the three measures of service leadership qualities, students showed significant improvements in knowledge (F ranged from 5.49 to 9.91, ps < 0.05, η^2^_p_ varied from 0.07 to 0.12) and behavior (F ranged from 9.93 to 15.16, ps < 0.01, η^2^_p_ ranged from 0.12 to 0.16) in all courses. For attitudinal measure, students showed significant improvement after taking the asynchronous online course in the 3rd Semester (F = 4.70, *p* < 0.05, η^2^_p_ = 0.06), but not the other two courses (F ≤ 1.81, *p* > 0.05). 

When combining the data obtained in three courses, omnibus time effects were significant for PYD attributes (F = 12.06, *p* < 0.001, η^2^_p_ = 0.18), life satisfaction (F = 44.15, *p* < 0.001, η^2^_p_ = 0.16) and leadership qualities (F = 18.42, *p* < 0.001, η^2^_p_ = 0.20). Further univariate analyses yielded positive changes in each PYD measure (η^2^_p_ varied between 0.10 and 0.16) and in the three indicators of service leadership qualities (η^2^_p_ ranged between 0.03 and 0.14). Based on these findings, we can conclude that students achieved significant improvements in their well-being and service leadership qualities after taking the “Service Leadership” course delivered through online learning modes. Besides, the course impact was not affected by the teaching mode or the course duration. Thus, both H1a and H1b were supported.

### 3.2. Profiles of Students’ Perceptions of the Subject 

Students’ positive perceptions of course content and lecturer performance are summarized in Table 3 and their positive responses toward course benefits are shown in Table 4. Regarding course content, the majority (i.e., over 75%) of students held positive perceptions on most of the evaluation items (e.g., objective of the subject, course design and learning experience). However, only around 56% of the students gave positive responses regarding peer interactions in the two asynchronous online courses. Nevertheless, over 92% of the students gave an overall positive evaluation on the course and nearly 85% of the students liked the course very much.

For lecturer performance, over 91% of the students in each course said that they had an overall positive perception on the lecturers. Specifically, more than 80% of the respondents had positive responses to all the rating items (e.g., preparation for the course, teaching attitudes and skills, caring for the students, and interactions with the students). For example, over 92% of the students in the three courses rated that the lecturers had good preparations for lessons and were willing to help students.

Regarding the perceived course benefits (Table 4), more than 86% of the students in each course agreed that the course was beneficial for their overall development. In particular, over 75% of the respondents perceived course benefits in almost all aspects, including social competence, emotional skills, critical thinking, resilience and self-leadership. In addition, over 90% of the students perceived that the course helped them “better understand and synthesize the characteristics of successful leaders” in the service economy. These findings provide support for H2a, H2b, and H2c.

The multivariate GLM analysis revealed a significant difference in the subjective evaluation across the three courses (Wilks’ λ = 0.89, F = 4.21, *p* < 0.001, η^2^_p_ = 0.06). Results of the follow-up univariate testes (see Table 5) suggested that there were significant differences on perceived course content (F = 7.71, *p* < 0.01, η^2^_p_ = 0.07) and lecturer performance (F = 9.22, *p* < 0.001, η^2^_p_ = 0.08), but not on course benefits (F = 2.99, *p* > 0.05, η^2^_p_ = 0.02). Further post-hoc tests revealed that students taking the synchronous online course in Semester 3 reported better evaluations of the course content (asynchronous online course in Semester 2: mean difference = 0.29, *p* < 0.01; asynchronous online course in Semester 3: mean difference = 0.22, *p* < 0.01) and lecturer performance (asynchronous online course in Semester 2: mean difference = 0.25, *p* < 0.01; asynchronous online course in Semester 3: mean difference = 0.30, *p* < 0.001) than did the students taking the other two courses. Students taking the two asynchronous online lectures did not show significant differences in their perceptions. 

### 3.3. Associations between Posttest Scores and Changes and Subjective Evaluations

Table 6 shows the correlations between posttest scores and changes (posttest score minus pretest score) in PYD attributes and well-being and their perceptions of the subject. In general, both posttest scores and student changes in total PYD score and life satisfaction were positively correlated with subjective outcome evaluation scores on course content, lecturer performance and course benefits, with correlation coefficients varying between 0.14 and 0.33 (ps < 0.05) for posttest scores and between 0.14 and 0.23 for changes (ps < 0.05). For service leadership qualities, changes in attitude and behavior, but not knowledge gain, were significantly associated with their perceptions on the course. However, the effect size was not large.

## 4. Discussion

Online learning, such as synchronous online learning and asynchronous learning that blends face-to-face and online instructions, has become increasingly popular in tertiary institutions [24,45]. For example, aiming to “join the best features of in-class teaching with the best features of online learning to promote active, self-directed learning opportunities for students” [46] (p. 82), blended learning has had a beneficial impact on student learning engagement, satisfaction and achievement [24,47]. However, there are also challenges for both teachers and students in taking the online learning mode, especially since the COVID-19 pandemic restricted face-to-face interactions and “forced” the overnight shift of in-person teaching to online teaching. Under these circumstances, the present study adds value to the extant literature by evaluating a leadership subject emphasizing experiential learning delivered through asynchronous or synchronous online modes in the 2019–2020 academic year during the COVID-19 pandemic. 

The one-group pretest-posttest evaluation revealed that students’ self-reported scores in the measures of service leadership qualities, subjective and psychological well-being significantly increased after taking the service leadership subject delivered in different online modes. The beneficial course effects are in line with previous positive evaluation findings for courses using face-to-face instructions [8,17]. Similar positive effects of online courses have also been reported before [48]. Taken together, the findings suggest that a meticulously designed subject that adopts experiential teaching pedagogy can be effectively delivered and well-received by students through online modes. In our case, students could interact with peers and teachers through diversified online activities that were adapted from face-to-face interactions. This helps to promote students’ engagement and active learning in the online courses, which are essential for achieving intended learning outcomes in online learning [49,50]. 

Furthermore, the two modes (i.e., asynchronous and synchronous) of online courses showed similar course effectiveness in the present study. Particularly, the asynchronous online course appeared to be more effective in promoting students’ attitudinal change than the synchronous one. These findings are inconsistent with a recent review, which concluded that synchronous online courses were more effective in promoting student learning than asynchronous ones [24,51]. Nevertheless, the effect size of the difference was trivial. Admittedly, as numerous factors may be related to online learning success [49,52], future studies need to verify the present findings and further investigate individual and contextual factors that may influence online learning. 

A large body of studies have reported elevated mental health issues and dampened well-being resulted from lockdown, school closures and difficulties in adapting to learning in virtual classrooms during COVID-19 pandemic [53,54,55]. Thus, the positive changes in students’ leadership qualities, PYD attributes and life satisfaction are quite encouraging and inspiring. Based on the present findings, online credit-bearing subjects could be a useful tool to help students deal with difficult situations. On the one hand, the highly encouraged peer interactions and collaborative learning may help lessen the negative influence of the pandemic such as lockdown and social distancing on students’ experience. On the other hand, it is possible that the positive thinking styles (e.g., leadership attributes can be cultivated), strength-based perspectives (e.g., every student has a potential to become an effective leader) and opportunities for reflection indeed enable students to deal with the current adversity using their strengths and translate the challenge into a gain in personal development. Meaningful engagement in learning and the gained competence development in a difficult situation allow students to enjoy a sense of achievement and positive feelings about themselves as well as their lives. Nevertheless, these possibilities need to be verified in future studies. For instance, qualitative evaluations can be carried out to understand possible reasons behind the observed improvement revealed by the quantitative findings. 

The positive changes in the students echo the positive subjective outcome evaluation findings that students generally reported positive perceptions about the course, the instructor and benefits regardless of the mode of the online course. This overall positive perception and satisfaction are in contrast with other research highlighting worries, concerns, distress and dissatisfaction with online learning during the pandemic [55,56]. When transforming the original service leadership subject into online courses, we adjusted teaching content and interactive activities to facilitate online learning, encouraged students to support each other and learn from each other and provided sufficient support (e.g., consultation sessions) for students throughout the learning process, all of which may help students adapt well to the new learning patterns and enhance their satisfaction with the learning journal. 

Despite the overall positive subjective evaluations, relatively lower percentages of students in the two asynchronous online courses considered peer interaction to be adequate, compared with students in the synchronous online course. Furthermore, comparisons of subjective evaluation scores indicated that the synchronous online course received more positive ratings on course content and teachers’ performance than did the two asynchronous online courses with small effect sizes. This finding echoes the previous findings of a negligibly higher level of satisfaction with real-time online instructions in comparison to asynchronous ones [24]. The trivial difference is reasonable given the lack of synchronous communication and immediate feedback in the asynchronous online course while the synchronous learning environment allows for real-time peer interactions in their group discussions or chat boxes and instant feedback from teachers given to students’ sharing or questions [57,58]. Nevertheless, students in the different online learning environments showed similar perceptions of the course benefits on their leadership capacities, PYD competence and life satisfaction. As many factors, such as peer interactions, student motivation and teacher support, may affect students’ subjective evaluations of online learning [59], future studies can further explore factors that contribute to learners’ satisfaction with the online learning environment.

As for the correlations of student posttest scores and changes to their subjective outcome perceptions, Kirkpatrick’s conceptual framework for learning evaluation suggests that reactions, such as satisfaction, facilitate learning achievement, resulting in a positive correlation between satisfaction and learning [32]. Inconsistent with this assumption, students’ knowledge acquisition was not significantly correlated with their subjective evaluations in our study. This result suggests that students’ learning achievement at the knowledge level may not have much to do with their affect (i.e., satisfaction). This is understandable as students’ knowledge learning might be driven by external motivation, such as passing the subject and earning the credits. This conjecture coincides with the previous findings showing no significant or even negative correlation between learners’ satisfaction and their learning success in terms of knowledge score [24,60]. However, in line with Kirkpatrick’s model, we found that improvement in students’ attitude, behavior and well-being was positively associated with satisfaction evaluations. This finding implies that students’ positive evaluation of the course (i.e., higher satisfaction) may inspire their intrinsic learning motivation and raise the likelihood of obtaining achievement beyond knowledge learning. Given the correlational relationship in nature, it is also possible that students’ learning achievement leads to their satisfaction with the online courses [49]. Future studies would benefit from conducting more longitudinal research to further explore the association between students’ learning achievement and subjective perception.

The current study has a few limitations. First of all, without an experimental design involving a control group, we are not able to conclusively attribute students’ improvement to their learning experience in the service leadership course. As students enrolled in the subject based on their own interest and schedule, random assignment of students into experimental or control groups is not feasible. Hence, future studies can adopt a quasi-experimental design that has been frequently used in the educational setting and involve students not taking the course as a control group [17,37]. Second, the study solely depended on self-reporting measures. For example, although the measure of service leadership behavior has been rigorously validated, it would also be methodologically superior to use other tools (e.g., diary and behavior checklist) to assess students’ actual leadership behavior in daily life. Finally, as the present study only evaluated the three online courses during COVID-19, it will be meaningful to compare the online courses with normal face-to-face teaching courses and further investigate factors contributing to the effectiveness of the online course.

## 5. Conclusions

The present study responded timely to the call for investigating course effectiveness of online teaching during COVID-19 and understanding students’ learning achievement and well-being during the pandemic. Our findings suggest that university students can have favorable learning experiences and evaluations in the adapted credit-bearing leadership subject delivered through asynchronous or synchronous online modes. They also gained significant improvement in their leadership capacity and well-being after completing the courses. These findings indicate that student learning in virtual classrooms can be as effective as in traditional physical classrooms. Accordingly, educators and researchers need to further consider how to effectively incorporate advanced technology in university education and further innovate leadership education pedagogy to fulfill a larger number of students’ learning needs under different situations.

## Figures and Tables

**Table 1:** Description of the matched sample in the pretest-posttest evaluation.

Variables		Semester 2 Asynchronous	Semester 3 Synchronous	Semester 3 Asynchronous	Total
Pretest-posttest evaluation					
N		75	79	74	228
Age	Mean	19.66	20.56	19.62	19.97
	SD	1.18	2.31	1.90	1.92
Gender	Males (n, %)	36 (48.00%)	35 (44.30%)	30 (40.54%)	101 (44.30%)
	Females (n, %)	39 (52.00%)	44 (55.70%)	44 (59.46%)	127 (55.70%)
Subjective outcome evaluation					
N		54	79	86	219
Age	Mean	19.76	20.56	19.61	20.01
	SD	1.09	2.31	1.87	1.94
Gender	Males (n, %)	25 (46.30%)	35 (44.30%)	35 (40.70%)	95 (43.38%)
	Females (n, %)	29 (53.70%)	44 (55.70%)	51 (59.30%)	124 (56.62%)

Note. SD = standard deviation; Asynchronous = ten asynchronous e-learning lectures + three synchronous online lectures; synchronous = 13 synchronous online lectures with in-class online interactions.

**Table 2:** Reliability and overall changes in different outcome indicators between the pretest and posttest.

Courses	Variables	Pretest		Posttest		F Value	η^2^_p_
		M (SD)	α (Mean ^#^)	M (SD)	α (Mean ^#^)		
Semester 2, Asynchronous mode, N = 75	Positive youth development					5.57 ***^,a^	0.24
	Cognitive-behavioral competence	4.50 (0.63)	0.87 (0.45)	4.73 (0.60)	0.88 (0.48)	14.50 ***	0.16
	Positive identity	4.33 (0.84)	0.84 (0.51)	4.62 (0.81)	0.89 (0.62)	15.48 ***	0.17
	General positive youth development qualities	4.53 (0.55)	0.87 (0.31)	4.62 (0.53)	0.86 (0.33)	2.69	0.04
	Total score of positive youth development qualities	4.48 (0.58)	0.94 (0.36)	4.65 (0.56)	0.93 (0.37)	11.03 **	0.09
	Life satisfaction	3.75 (1.06)	0.91 (0.67)	4.17 (1.02)	0.92 (0.69)	15.80 ***	0.18
	Service leadership qualities					5.78 **^,b^	0.20
	Service leadership knowledge	29.23 (9.81)	0.91 (0.38)	31.84 (8.60)	0.94 (0.30)	5.49 *	0.07
	Service leadership attitude	4.93 (0.45)	0.92 (0.39)	5.00 (0.64)	0.95 (0.54)	1.50	0.02
	Service leadership behavior	4.62 (0.52)	0.94 (0.31)	4.81 (0.55)	0.84 (0.41)	11.26 **	0.13
Semester 3, Synchronous mode, N = 79	Positive youth development					7.16 ***^,a^	0.22
	Cognitive-behavioral competence	4.54 (0.59)	0.87 (0.42)	4.85 (0.52)	0.89 (0.47)	19.90 ***	0.20
	Positive identity	4.41 (0.83)	0.85 (0.54)	4.72 (0.75)	0.88 (0.60)	16.03 ***	0.17
	General positive youth development qualities	4.56 (0.48)	0.83 (0.27)	4.76 (0.52)	0.84 (0.32)	15.89 ***	0.17
	Total score of positive youth development qualities	4.52 (0.53)	0.92 (0.31)	4.78 (0.52)	0.93 (0.36)	21.69 ***	0.22
	Life satisfaction	3.88 (0.93)	0.86 (0.56)	4.22 (0.96)	0.91 (0.67)	10.14 **	0.12
	Service leadership qualities					6.94 ***^,b^	0.22
	Service leadership knowledge	28.00 (10.00)	0.95 (0.31)	30.30 (10.01)	0.96 (0.37)	7.79 **	0.09
	Service leadership attitude	4.93 (0.48)	0.94 (0.42)	5.00 (0.54)	0.94 (0.49)	1.81	0.02
	Service leadership behavior	4.73 (0.55)	0.95 (0.48)	4.96 (0.53)	0.96 (0.59)	15.16 **	0.16
Semester 3, Asynchronous mode, N = 74	Positive youth development					4.72 **^,a^	0.17
	Cognitive-behavioral competence	4.54 (0.65)	0.92 (0.57)	4.70 (0.70)	0.93 (0.59)	4.35 *	0.06
	Positive identity	4.23 (0.79)	0.88 (0.61)	4.56 (0.81)	0.92 (0.90)	12.71 ***	0.15
	General positive youth development qualities	4.42 (0.57)	0.83 (0.25)	4.60 (0.64)	0.90 (0.42)	9.08 **	0.11
	Total score of positive youth development qualities	4.42 (0.58)	0.92 (0.30)	4.62 (0.68)	0.96 (0.49)	10.19 **	0.12
	Life satisfaction	3.81 (1.03)	0.93 (0.73)	4.28 (1.01)	0.92 (0.71)	18.88 **	0.21
	Service leadership qualities					5.81 **^,b^	0.20
	Service leadership knowledge	29.45 (9.02)	0.93 (0.25)	32.23 (9.15)	0.95 (0.36)	9.91 **	0.12
	Service leadership attitude	4.80 (0.61)	0.95 (0.49)	4.95 (0.66)	0.96 (0.59)	4.70 *	0.06
	Service leadership behavior	4.58 (0.66)	0.95 (0.52)	4.80 (0.67)	0.96 (0.58)	9.93 **	0.12

Note. ^#^ Mean inter-item correlations. ^a^ Adjusted Bonferroni value = 0.013. ^b^ Adjusted Bonferroni value = 0.017. * *p* < 0.05. ** *p* < 0.01. *** *p* < 0.001.

**Table 3:** Positive responses (options 4–5) regarding participants’ evaluations on the course content and lecturer performance.

Items	Semester 2 Asynchronous		Semester 3 Synchronous		Semester 2 Asynchronous		Total	
	*n*	%	*n*	%	*n*	%	*n*	%
Course Content								
1. The objectives of the curriculum are very clear.	40	75.47	74	94.87	80	93.02	194	89.40
2. The content design of the curriculum is very good.	48	90.57	72	91.14	79	91.86	199	91.28
3. The activities were carefully arranged.	49	92.45	72	91.14	77	89.53	198	90.83
4. The (virtual) classroom atmosphere was very pleasant.	40	74.07	73	92.41	69	80.23	182	83.11
5. There was much peer interaction amongst the students.	32	59.26	69	87.34	49	56.98	150	68.49
6. I participated in the class activities actively (including discussions, sharing, games, etc.).	41	75.93	65	82.28	66	76.74	172	78.54
7. I was encouraged to do my best.	47	87.04	70	88.61	68	79.07	185	84.47
8. The learning experience enhanced my interests towards the course.	45	83.33	67	85.90	72	83.72	184	84.40
9. Overall speaking, I have a very positive evaluation on the course.	50	92.59	73	92.41	80	93.02	203	92.69
10. On the whole, I like this course very much.	46	85.19	71	91.03	73	84.88	190	87.16
Lecturer Performance								
1. The lecturer(s) had a good mastery of the course.	49	90.74	76	96.20	80	93.02	205	93.61
2. The lecturer(s) was (were) well prepared for the lessons.	50	92.59	78	98.73	81	94.19	209	95.43
3. The teaching skills of the lecturer(s) were good.	46	85.19	75	94.94	69	80.23	190	86.76
4. The lecturer(s) showed good professional attitudes.	51	94.44	78	98.73	78	90.70	207	94.52
5. The lecturer(s) was (were) very involved.	48	88.89	79	100.00	79	91.86	206	94.06
6. The lecturer(s) encouraged students to participate in the activities.	50	94.34	79	100.00	76	89.41	205	94.47
7. The lecturer(s) cared for the students.	50	92.59	73	92.41	72	84.71	195	89.45
8. The lecturer(s) was (were) ready to offer help to students when needed.	53	98.15	77	97.47	80	93.02	210	95.89
9. The lecturer(s) had much interaction with the students.	47	87.04	76	96.20	64	74.42	187	85.39
10. Overall speaking, I have a very positive evaluation on the lecturer(s).	51	94.44	77	97.47	79	91.86	207	94.52

Note. All items were rated on a 5-point Likert scale with 1 = strongly disagree, 2 = disagree, 3 = neutral, 4 = agree, 5 = strongly agree. Only the positive responses (options 4–5) are shown.

**Table 4:** Respondents with positive responses (options 4–5) regarding participants’ perceived course benefits.

Items	Semester 2 Asynchronous		Semester 3 Synchronous		Semester 2 Asynchronous		Total	
	*n*	%	*n*	%	*n*	%	*n*	%
1. It has enhanced my social competence.	47	87.04	71	89.87	71	82.56	189	86.30
2. It has improved my ability in expressing and handling my emotions.	47	88.68	71	91.03	72	83.72	190	87.56
3. It has enhanced my critical thinking.	48	88.89	73	92.41	73	84.88	194	88.58
4. It has increased my competence in making sensible and wise choices.	48	88.89	70	88.61	72	83.72	190	86.76
5. It has helped me make ethical decisions.	45	83.33	74	93.67	77	91.67	196	90.32
6. It has strengthened my resilience in adverse conditions.	47	87.04	68	87.18	76	88.37	191	87.61
7. It has strengthened my self-confidence.	44	81.48	67	84.81	64	74.42	175	79.91
8. It has helped me face the future with a positive attitude.	45	83.33	71	89.87	73	84.88	189	86.30
9. It has enhanced my love for life.	40	75.47	64	81.01	65	75.58	169	77.52
10. It has helped me explore the meaning of life.	44	81.48	66	83.54	66	76.74	176	80.37
11. It has enhanced my ability of self-leadership.	46	86.79	76	97.44	78	90.70	200	92.17
12. It has helped me cultivate compassion and care for others.	46	85.19	72	92.31	73	84.88	191	87.61
13. It has helped me enhance my character strengths comprehensively.	47	87.04	76	97.44	77	89.53	200	91.74
14. It has enabled me to understand the importance of situational task competencies, character strength and caring disposition in successful leadership.	49	90.74	76	96.20	79	91.86	204	93.15
15. It has promoted my sense of responsibility in serving the society.	48	88.89	70	88.61	73	84.88	191	87.21
16. It has promoted my overall development.	46	86.79	76	96.20	76	88.37	198	90.83
17. The theories, research and concepts covered in the course have enabled me to understand the characteristics of successful service leaders.	49	92.45	77	97.47	78	90.70	204	93.58
18. The theories, research and concepts covered in the course have helped me synthesize the characteristics of successful service leaders.	50	92.59	77	98.72	80	93.02	207	94.95

Note. All items were rated on a 5-point Likert scale with 1 = unhelpful, 2 = not very helpful, 3 = not sure, 4 = helpful, 5 = strongly agree. Only positive responses (options 4–5) are shown.

**Table 5:** Comparisons of subjective evaluation among three courses.

Evaluations	Semester 2 Asynchronous		Semester 3 Synchronous		Semester 3 Asynchronous		Comparison	
	M	SD	M	SD	M	SD	F Value	Partial η^2^
Course content	3.95	0.38	4.25	0.49	4.03	0.46	7.71 **	0.07
Lecturer performance	4.21	0.43	4.46	0.45	4.17	0.50	9.22 ***	0.08
Course benefits	4.06	0.47	4.21	0.51	4.04	0.46	2.99	0.02

Note. M = Mean, SD = standard deviation, Partial η^2^ indicates effect size. ** *p* < 0.01. *** *p* < 0.001.

**Table 6:** Correlations between students’ subjective outcome evaluations and their posttest scores, as well as changes after taking the course (posttest score minus pretest score) (N = 207).

Objective Outcome Measures	Perception on Course Content		Perception on Lecturer Performance		Perception on Course Effect	
	Posttest	Changes	Posttest	Changes	Posttest	Changes
Cognitive-behavioral competence	0.29 ***	0.21 **	0.21 **	0.21 **	0.26 ***	0.17 *
Positive identity	0.27 ***	0.14 *	0.14 *	0.13	0.27 ***	0.11
General PYD qualities	0.33 ***	0.22 **	0.22 **	0.18 **	0.29 ***	0.17 *
Total score of PYD qualities	0.32 ***	0.22 **	0.21 **	0.20 **	0.29 ***	0.17 *
Life satisfaction	0.27 ***	0.23 **	0.17 *	0.15 *	0.30 ***	0.21 **
Service leadership knowledge	−0.01	0.05	−0.08	0.05	−0.06	−0.05
Service leadership attitude	0.25 ***	0.16 *	0.19 **	0.16 *	0.16 *	0.08
Service leadership behavior	0.35 ***	0.21 **	0.25 ***	0.22 **	0.34 ***	0.19 **

* *p* < 0.05. ** *p* < 0.01. *** *p* < 0.001.

## Data Availability

The data presented in this study are available on request from the corresponding author. The data are not publicly available due to privacy.
